# Supplementary figures and images for: Enhancing CRISPR prime editing by reducing misfolded pegRNA interactions
Source: eLife. 2024 Jun 7;12:RP90948. doi: 10.7554/eLife.90948 (PMC11161173; doi:10.7554/eLife.90948)

a

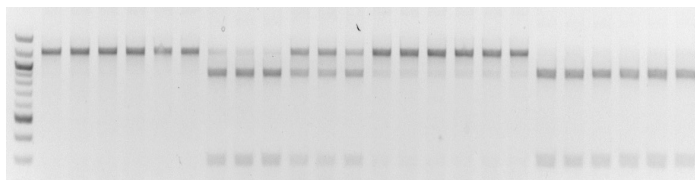

b

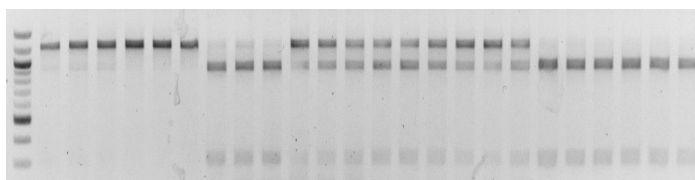

c

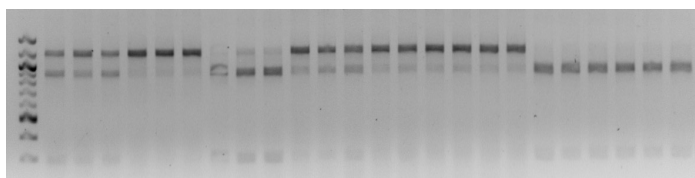

d

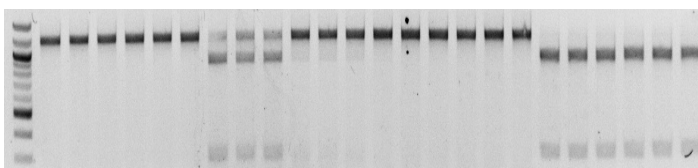

Supplement: Figure 1—source data 1. [file elife-90948-fig1-data1.pdf]

**a** *adgrf3b*

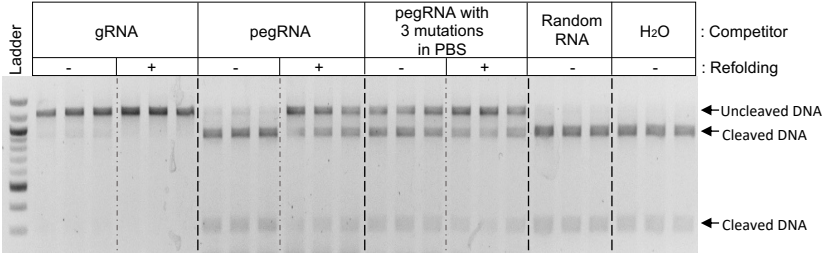

**b** *cacng2b*

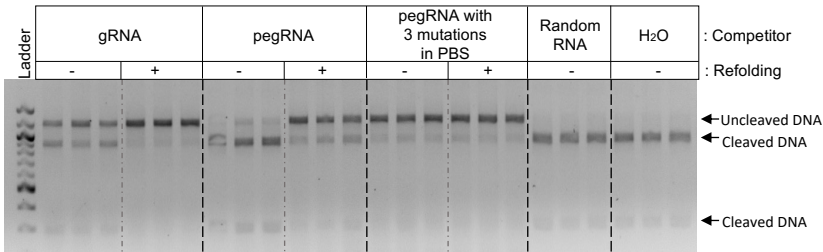

**c** *gpr85*

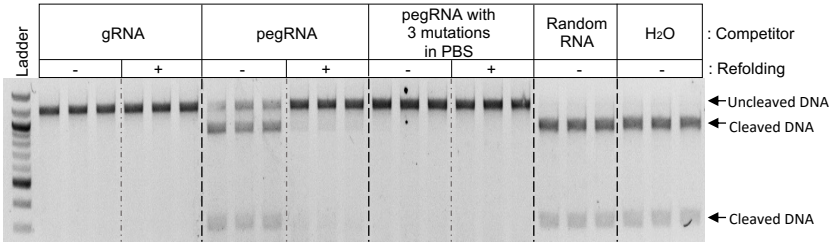

Supplement: Figure 1—source data 2. — The percentage of uncleaved DNA substrate in the presence or absence of competitor gRNA or pegRNA calculated using this data is shown in Figure 1f. [file elife-90948-fig1-data2.pdf]
