# Supplementary material for: Enhancing CRISPR prime editing by reducing misfolded pegRNA interactions
Source: eLife. 2024 Jun 7;12:RP90948. doi: 10.7554/eLife.90948 (PMC11161173; doi:10.7554/eLife.90948)
Supplement: Figure 1—source data 3. — The RT template (RTT) sequences are shown in red. The PBS sequences are underlined. The three mutations in PBS are shown in orange. [file elife-90948-fig1-data3.pdf]

pegRNA	pegRNA 5' spacer sequence (5'->3')	pegRNA 3' extension sequence for RTT and PBS (5'->3')	Specified edit	PBS length (nucleotides)	RTT length (nucleotides)
gpr78a-P10R14-S5-C9E	GAAACACTACGGCTATCCAG	GGCATAGACAGCTGGATAGCCGTA	+5 G->T	10	14
gpr78a-P10R14-S5-C9E-3MUTSinPBS	GAAACACTACGGCTATCCAG	GGCATAGACAGCTGCATTGCGGTA	+5 G->T	10	14
adgrf3b-P10R15-S5-C9E	GGTGTATGGCAGATGTCCAG	GCTTCGTCGCGTCTGGACATCTGCC	+5 G->C	10	15
adgrf3b-P10R15-S5-C9E-3MUTSinPBS	GGTGTATGGCAGATGTCCAG	GCTTCGTCGCGTCTGCACTTCAGCC	+5 G->C	10	15
cacng2b-P10R15-S5-C9E	GGTGGAATGCGGCAGTATGG	GCTCAAACACGCCCATACTGCCGCA	+5 G->C	10	15
cacng2b-P10R15-S5-C9E-3MUTSinPBS	GGTGGAATGCGGCAGTATGG	GCTCAAACACGCCCAAACAGCGGCA	+5 G->C	10	15
gpr85-P10R14-S5-C9E	GGAGGTCAGTTTAAGGAAAG	TCCGCTCGCGACTTTCCTTAAACT	+5 G->C	10	14
gpr85-P10R14-S5-C9E-3MUTSinPBS	GGAGGTCAGTTTAAGGAAAG	TCCGCTCGCGACTTACCATATACT	+5 G->C	10	14
